# Machine Learning Approaches to Predict Hepatotoxicity Risk in Patients Receiving Nilotinib

**DOI:** 10.3390/molecules26113300

**Published:** 2021-05-31

**Authors:** Jung-Sun Kim, Ji-Min Han, Yoon-Sook Cho, Kyung-Hee Choi, Hye-Sun Gwak

**Affiliations:** 1 College of Pharmacy, Graduate School of Pharmaceutical Sciences, Ewha Womans University, Seoul 03760, Korea; 30607@snuh.org; 2 Department of Pharmacy, Seoul National University Hospital, Seoul 03080, Korea; joys99@snuh.org; 3 College of Pharmacy, Chungbuk National University, Cheongju-si 28160, Korea; jmhan@chungbuk.ac.kr; 4 College of Pharmacy, Sunchon National University, Suncheon 57922, Korea; khchoi@sunchon.ac.kr

**Keywords:** nilotinib, hepatotoxicity, male, H2 blocker, dose, machine learning

## Abstract

**Background:** Although nilotinib hepatotoxicity can cause severe clinical conditions and may alter treatment plans, risk factors affecting nilotinib-induced hepatotoxicity have not been investigated. This study aimed to elucidate the factors affecting nilotinib-induced hepatotoxicity. **Methods:** This retrospective cohort study was performed on patients using nilotinib from July of 2015 to June of 2020. We estimated the odds ratio and adjusted odds ratio from univariate and multivariate analyses, respectively. Several machine learning models were developed to predict risk factors of hepatotoxicity occurrence. The area under the curve (AUC) was analyzed to assess clinical performance. **Results:** Among 353 patients, the rate of patients with grade I or higher hepatotoxicity after nilotinib administration was 40.8%. Male patients and patients who received nilotinib at a dose of ≥300 mg had a 2.3-fold and a 3.5-fold increased risk for hepatotoxicity compared to female patients and compared with those who received <300 mg, respectively. H2 blocker use decreased hepatotoxicity by 11.6-fold. The area under the curve (AUC) values of machine learning methods ranged between 0.61–0.65 in this study. **Conclusion:** This study suggests that the use of H2 blockers was a reduced risk of nilotinib-induced hepatotoxicity, whereas male gender and a high dose were associated with increased hepatotoxicity.

## 1. Introduction

Nilotinib is a second-generation tyrosine kinase Bcr-Abl (c-Abl) inhibitor [1]. Since it showed improved efficacy and selectivity for c-Abl over imatinib, it was initially approved for the treatment of patients with imatinib-resistant/intolerant or newly diagnosed chronic myelogenous leukemia [2,3]. Nilotinib has similar activity to imatinib, including its potency against the discoidin domain receptor, stem cell factor receptor, platelet-derived growth factor receptor, and colony-stimulating factor receptor-1 [4,5]. 

The efficacy of nilotinib has been evaluated in neurodegenerative diseases, such as Parkinson’s disease, Alzheimer’s disease, and cerebellar ataxia. In a mouse model, c-Abl activation was related to neurodegeneration in the hippocampus and striatum [6]. Several animal models of neurodegeneration demonstrated that nilotinib degrades misfolded alpha-synuclein in Lewy bodies by inhibiting the discoidin domain receptor and decreasing a tau level, which is related to cognitive decline in Alzheimer’s disease [7,8,9,10]. Increased dopamine levels and functional improvements in Parkinson’s disease, Alzheimer’s disease, and cerebellar ataxia have been confirmed [11,12,13]. 

Nilotinib is well-tolerated in clinical trials [14,15,16]. Common toxicities of nilotinib include transient skin rash, headache, nausea, pruritus, and myalgia [17]. Elevated aspartate aminotransferase (AST) and alanine aminotransferase (ALT) were also reported as frequent adverse drug reactions [18]. Severe cases, including grade III-IV hepatotoxicity, were infrequent (1–4%) [1,19,20]; however, patients who experienced hepatotoxicity while taking nilotinib had aggravated clinical conditions that could affect their treatment plan [21,22]. 

Risk factors affecting nilotinib-induced hepatotoxicity have not been investigated. Thus, the purpose of this study was to elucidate risk factors for nilotinib-induced hepatotoxicity and use supervised machine learning methods to build predictive models of hepatotoxicity occurrence. 

## 2. Results

A total of 497 patients treated from July 2015 to June 2020 were eligible for participation in this study. We excluded patients who had elevated AST and ALT before nilotinib administration (*n* = 48) and those who had inappropriate follow-up data after nilotinib administration such as no liver function tests at the initiation of nilotinib or after nilotinib administration (*n* = 96). Data from 353 patients treated with nilotinib were included for the analysis. 

As shown in Table 1, 169 patients (47.9%) were older than 60 years (age range, 20–92 years). Approximately 51% of the study patients were male, and 144 patients (40.8%) experienced hepatotoxicity after nilotinib administration. Drugs concurrently administered with nilotinib included anticancer drugs (*n* = 5), acetaminophen (*n* = 3), 3-hydroxy-3-methyl-glutaryl-coenzyme A (HMG-CoA) reductase inhibitors (*n* = 58), antihypertensives (*n* = 49), immunosuppressants (*n* = 5), cytochrome P450 (CYP) 3A4 inhibitors (*n* = 7), CYP3A4 inducers (*n* = 15), H2 blockers (*n* = 22), and proton pump inhibitors (PPIs) (*n* = 4). In the univariate analysis, sex, presence of cardiovascular disease (CVD) or diabetes mellitus (DM), and a daily nilotinib dose of ≥300 mg were significant factors for hepatotoxicity.

**Table 1:** Hepatotoxicity related to nilotinib administration.

Characteristics		No. (%)	Hepatotoxicity, No (%)		*p*
		(*n* = 353)	Absence	Presence	
			(*n* = 209)	(*n* = 144)	
Sex	Female	172 (48.7)	115 (55.0)	57 (39.6)	0.004
	Male	181 (51.3)	94 (45.0)	87 (60.4)	
Age, years	<60	184 (52.1)	113 (54.1)	71 (49.3)	0.379
	≥60	169 (47.9)	96 (45.9)	73 (50.7)	
Body weight ^a^, kg	<65	83 (57.2)	35 (57.4)	48 (57.1)	0.978
	≥65	62 (42.8)	26 (42.6)	36 (42.9)	
Height ^b^, cm	<163	66 (46.5)	31 (50.8)	35 (43.2)	0.368
	≥163	76 (53.5)	30 (49.2)	46 (56.8)	
BSA ^b^, m^2^	<1.7	80 (56.3)	37 (60.7)	43 (53.1)	0.368
	≥1.7	62 (43.7)	24 (39.3)	38 (46.9)	
Alcohol history ^c^	Yes	17 (13.9)	4 (7.5)	13 (18.8)	0.074
	No	105 (86.1)	49 (92.5)	56 (81.2)	
CVD or DM ^d^	Yes	98 (27.8)	50 (23.9)	48 (33.6)	0.047
	No	254 (72.2)	159 (76.1)	95 (66.4)	
Daily dose, mg	<300	73 (20.7)	56 (26.8)	17 (11.8)	0.001
	≥300	280 (79.3)	153 (73.2)	127 (88.2)	
Anticancer drugs ^d^	Yes	5 (1.4)	2 (1.0)	3 (2.1)	0.382
	No	347 (98.6)	206 (99.0)	141 (97.9)	
Acetaminophen ^d^	Yes	3 (0.9)	2 (1.0)	1 (0.7)	0.789
	No	349 (99.1)	206 (99.0)	143 (99.3)	
HMG-coA reductase inhibitors ^d^	Yes	58 (16.5)	33 (15.9)	25 (17.4)	0.710
	No	294 (83.5)	175 (84.1)	119 (82.6)	
Antihypertensives ^d^	Yes	49 (13.9)	31 (14.9)	18 (12.5)	0.522
	No	303 (86.1)	177 (85.1)	126 (87.5)	
Immunosuppressants ^d^	Yes	5 (1.4)	1 (0.5)	4 (2.8)	0.073
	No	347 (98.6)	207 (99.5)	140 (97.2)	
CYP3A4 inhibitors ^d^	Yes	7 (2.0)	4 (1.9)	3 (2.1)	0.916
	No	345 (98.0)	204 (98.1)	141 (97.9)	
CYP3A4 inducers ^d^	Yes	15 (4.3)	6 (2.9)	9 (6.3)	0.124
	No	337 (95.7)	202 (97.1)	135 (93.8)	
H2 blockers ^d^	Yes	22 (6.3)	17 (8.2)	5 (3.5)	0.073
	No	330 (93.8)	191 (91.8)	139 (96.5)	
PPIs ^d^	Yes	4 (1.1)	2 (1.0)	2 (1.4)	0.710
	No	348 (98.9)	206 (99.0)	142 (98.6)	

BSA, body surface area; CVD, cardiovascular disease; CYP3A4 inducer, cytochrome P450 3A4 inducer; CYP3A4 inhibitor, cytochrome P450 3A4 inhibitor; DM, diabetes mellitus; HMG-coA reductase inhibitor, 3-hydroxy-3-methyl-glutaryl-coenzyme A (HMG-CoA) reductase inhibitors; ^a^ There were 208 missing data for body weight. ^b^ There were 211 missing data for height and BSA. ^c^ There were 231 missing data for alcohol history. ^d^ There was 1 missing data for CVD or DM, anticancer drugs, acetaminophen, HMG-coA reductase inhibitors, antihypertensives, immunosuppressants, CYP3A4 inhibitors, CYP3A4 inducers, H2 blockers, and PPIs. After adjusting for factors with *p* < 0.1 in the univariate analysis, male patients had 2.3-fold increased hepatotoxicity risk compared with female patients (Table 2). A daily nilotinib dose of 300 mg or greater increased hepatotoxicity incidence by 3.5-fold. Concomitant use of H2 blocker decreased the risk of hepatotoxicity by 11.6 times.

Since feature importance by random forest (RF) was ranked in the order of daily dose (5.30) > sex (4.19) > CVD or DM (3.10) > age (2.64) > H2 blockers (2.61) > alcohol history (0.80) > immunosuppressants (0.33), daily dose, sex, CVD or DM, age, and H2 blockers were used for machine learning. After performing a 5-fold cross-validated multivariate logistic regression, elastic net, RF, and support vector machine (SVM) models (linear kernel and radial kernel), the average area under the receiver-operating curve (AUROC) values were 0.65, 0.65, 0.63, 0.61, and 0.64, respectively (Table 3). 

## 3. Discussion

This study demonstrated that male sex and a nilotinib daily dosage of ≥300 mg were significant factors for hepatotoxicity incidence. Male patients had a 2.3-fold higher risk for hepatotoxicity than females. Patients who received nilotinib at a dose of ≥300 mg had 3.5 times the risk for hepatotoxicity compared with those who received < 300 mg. H2 blockers decreased hepatotoxicity by 11.6-fold. Five-fold cross-validated multivariate logistic regression, elastic net, RF, and SVM models had AUROC values across 100 random iterations that ranged between 0.61–0.65.

The mechanism of drug-induced hepatotoxicity is heterogeneous, including direct hepatotoxicity, immune-mediated liver injury, and mitochondria-selective toxicity [23]. Nilotinib hepatotoxicity has an unclear mechanism, but it may be immune-related [22]. A case report regarding nilotinib-induced hepatotoxicity demonstrated an infiltration of killer cells, such as cytotoxic T (CD8+ T) cells and natural killer cells, in a patient’s liver biopsy [22]. 

Females are usually more susceptible to adverse drug reactions than males [24]. In contrast, our study showed that male patients had an increased risk of hepatotoxicity compared to female patients. This may be due to the male patients’ history of frequent alcohol use. Due to a lack of records, it was not possible to obtain an alcohol history from all patients, which resulted in no statistical significance. However, the proportion of male patients among all patients with a history of alcohol use was 88% (15 out of 17 patients), and 12 of these (80%) experienced hepatotoxicity. Considering that the ratio of male to female patients in our study was almost 1:1, alcohol intake could be one of the confounding factors affecting male sex being a risk factor for nilotinib-induced hepatotoxicity.

Doses larger than 300 mg resulted in a significantly higher incidence of hepatotoxicity. In a population pharmacokinetic study, patients receiving nilotinib showed different levels of exposure depending on the dose [25]. Patients receiving 400 mg of nilotinib twice daily had a higher exposure than patients receiving 300 mg of nilotinib twice daily. The incidence of total bilirubin elevation in all grades was also significantly higher. This result is consistent with our findings that the risk of nilotinib-induced hepatotoxicity increased at higher doses. 

The absorption of many oral tyrosine kinase inhibitors (TKIs) is affected by gastric pH. Nilotinib also exhibits pH-dependent solubility, exhibiting decreased solubility with increasing pH. Nilotinib is insoluble in buffer solutions above a pH of 4.5 [26]. It has been suggested that there are possible interactions between nilotinib and anti-acid secreting agents, such as PPIs and H2 blockers, which can elevate gastric pH. In a previous pharmacokinetic study that investigated interactions between nilotinib and esomeprazole, the area under the curve (AUC) of nilotinib was reduced by 34% when concurrently administered with esomeprazole [27]. Thus, the concurrent use of other anti-acid secreting agents such as H2-blocker can decrease nilotinib absorption and reduce the incidence of adverse drug events, including hepatotoxicity. 

ATP-binding cassette subfamily B member 1 (ABCB1) and ATP-binding cassette superfamily G member 2 (ABCG2), drug efflux transporters, are located in various normal tissues, including the liver [28]. Several studies have demonstrated that these transporters interact with nilotinib [29,30,31]. In the case of the ABCB1 transporter, nilotinib acts as a substrate at low concentrations and as an inhibitor at high concentrations. The study that reported nilotinib as an inhibitor used an extremely high concentration, and so nilotinib may function as a substrate at clinical concentrations [32]. Since the H2 blocker is also an ABCB1 substrate, co-administration of the H2 blocker and nilotinib can lead to competitive efflux out of the liver, thereby increasing the drug concentration in the liver. 

Our previous studies using gefitinib and erlotinib revealed that the concurrent use of an H2 blocker increased hepatotoxicity risk [33,34]. However, this study had the opposite results. This may be attributable to nilotinib’s poor solubility and permeability. Nilotinib is a biopharmaceutics classification system (BSC) class IV compound, indicating that it has poor bioavailability, while other studied TKIs are classified as BSC class II [26,35,36]. Therefore, we speculate that the amount of nilotinib in the liver was very low due to decreased absorption in addition to low permeability and solubility. 

Since the PPI is not an anti-secreting agent and an ABCG2 inhibitor, its concurrent use with nilotinib may alter hepatotoxicity risk. However, the PPI was not a significant factor for hepatotoxicity because of the small sample size (four patients). 

In this study, various machine learning approaches were utilized to predict nilotinib-induced hepatotoxicity. The AUC value from the 5-fold cross-validated multivariable logistic regression model was the same as that from the elastic net, a penalized linear regression model that combines penalties of the lasso and ridge methods [37]. Meanwhile, RF is an ensemble method that grows binary classification trees based on bootstrapped samples of the training data while using only a random subset of available features at each node to find the optimal splitting rule [38,39,40]. By repeating these processes, RF can generate thousands of decorrelated decision trees (i.e., the ensemble) that can provide more robust committee-type decisions. SVMs were implemented using linear and radial basis function kernels in this study. Linear kernel SVMs have a single tuning parameter C, the cost parameter of the error term, whereas radial kernel SVMs have an additional hyperparameter that defines the variance of the Gaussian, i.e., how far a single training example’s radius of influence reaches [40,41].

One limitation of this study is its retrospective design. Due to the lack of dietary information, it was not possible either to calculate or adjust for confounders. Moreover, other factors that may affect machine learning model performance including external validation must be considered. Nevertheless, this is the first study to investigate the risk factors for nilotinib-induced hepatotoxicity. This study utilized machine learning approaches to predict hepatotoxicity occurrence. Our results contribute to clinical decision-making for nilotinib-induced hepatotoxicity. 

In conclusion, our study showed that the male sex and ≥ 300 mg of nilotinib were associated with hepatotoxicity, while the use of H2 blocker was related to a decreased risk of hepatotoxicity. Since this was a retrospective study, further validation with a larger, prospective study is required. 

## 4. Materials and Methods

### 4.1. Patients

This study was performed from July of 2015 to June of 2020 at the Seoul National University Hospital in Korea. Eligible patients were individuals older than 18 years of age who received nilotinib to treat hematologic malignancies and neurodegenerative disorders such as cerebellar ataxia. Patients who already had elevated AST or ALT levels before nilotinib administration and those who had inappropriate follow-up data after nilotinib administration were excluded. There were no patients who had underlying liver disease. 

We collected the following demographic and clinical data: sex, age, height, body weight, body surface area, alcohol history, underlying CVD or DM, nilotinib daily dose, and concomitant medications. Concomitant medications included anticancer drugs, acetaminophen, HMG-CoA reductase inhibitors, antihypertensives, immunosuppressants, CYP3A4 inhibitors, CYP3A4 inducers, H2 blockers, and PPIs. Anticancer drugs included methotrexate, cytarabine, hydroxyurea, gemcitabine, and cisplatin. HMG-CoA reductase inhibitors included pitavastatin, atorvastatin, rosuvastatin, simvastatin, and fluvastatin. Antihypertensives included amlodipine, olmesartan, valsartan, fimasartan, nebivolol, atenolol, carvedilol, candesartan, telmisartan, losartan, bisoprolol, and propranolol. Immunosuppressants included tacrolimus, mycophenolate mofetil, and azathioprine. CYP3A4 inhibitors included diltiazem, amiodarone, oxcarbazepine, allopurinol, valproic acid, verapamil, fluconazole, and isoniazid. CYP3A4 inducers included carbamazepine, corticosteroids, clarithromycin, and phenobarbital. H2 blockers included ranitidine, famotidine, and nizatidine. PPIs included pantoprazole, esomeprazole, and lansoprazole.

### 4.2. Nilotinib Administration and Laboratory Assessment

Patients received nilotinib (150 to 800 mg/day) orally. A liver function test was performed before therapy initiation and every two to three months thereafter. We obtained serum AST and serum ALT levels and determined the hepatotoxicity grade using the Common Terminology Criteria for Advanced Events (CTCAE), version 4.0. CTCAE defines grade I toxicity levels of AST and ALT as 1.0–3.0 times the upper limit of normal, grade II as 3.0–5.0 times, grade III as 5.0–20.0 times, and grade IV as more than 20 times. In this study, hepatotoxicity was defined as grade I or higher. 

### 4.3. Statistical Analysis and Machine Learning Methods

A chi-squared or Fisher’s exact test was performed to compare categorical variables between patients with and without hepatotoxicity. A multivariate logistic regression analysis was performed to identify independent risk factors for hepatotoxicity. Factors having *p* < 0.1 from the univariate analysis along with strong confounders of sex and age were included in the multivariate analysis. Odds ratio (OR) and adjusted OR were estimated from the univariate and multivariate analyses, respectively. 

Machine learning models were developed to predict the risk factors of hepatotoxicity occurrence. RF was utilized for the selection of features among variables with *p* value less than 0.1 in the univariate analysis. Classification models, such as 5-fold cross-validated multivariable logistic regression, elastic net, RF, and SVM, were utilized. All methods were implemented with an R package caret. For cross-validation, the dataset was randomly divided into five equal folds. After partitioning one data sample into five subsets, one subset was selected for model validation while the remaining subsets were used to establish machine learning models. Each cross-validation iteration was repeated 100 times to evaluate the power of the machine learning models.

We analyzed the AUROC and sensitivity/specificity of each model to assess their ability to predict hepatotoxicity. A *p* < 0.05 was considered statistically significant. Univariate and multivariate analyses were performed with the Statistical Package for Social Sciences (SPSS) version 20.0 for Windows (IBM Corp., Armonk, NY). Machine learning models were constructed using R software version 3.6.0 (RFoundation for Statistical Computing, Vienna, Austria).

## Figures and Tables

**Table 2:** Univariate and multivariate analyses to identify predictors for hepatotoxicity related to nilotinib administration.

Characteristics		Unadjusted OR	Adjusted OR
		(95% CI)	(95% CI)
Male		1.867(1.213–2.874) *	2.293(1.051–4.999) *
Age, years	≥60	1.210(0.791–1.851)	
CVD or DM		1.607(1.004–2.572) *	
Alcohol history		2.844(0.870–9.296)	
Daily dose, mg	≥300	2.734(1.513–4.940) *	3.541(1.388–8.578) **
Immunosuppressants		5.914(0.654–53.471)	
H2 blockers		0.404(0.146–1.122)	0.086(0.009–0.791) *

CVD, cardiovascular disease; DM, diabetes mellitus; OR, odds ratio, * *p* < 0.05, ** *p* < 0.01.

**Table 3:** Machine learning models’ performance.

Model	AUROC	Sensitivity	Specificity
Multivariate logistic regression	0.65	85.9	26.9
Elastic net	0.65	90.9	22.4
Random forest	0.63	75.2	38.2
Support vector machine (linear)	0.61	90.0	13.9
Support vector machine (radial)	0.64	63.0	32.9

AUROC, area under the receiver-operating curve.

## Data Availability

The data presented in this study are available on request from the corresponding author. The data are not publicly available due to privacy and ethical reason.

## Sample Availability

Samples of the compounds are not available from the authors.
